# Investigation of the biocompatibility of various pulp capping materials on zebrafish model

**DOI:** 10.1371/journal.pone.0310996

**Published:** 2024-09-20

**Authors:** Meltem Karahan, Bahar Basak Kiziltan Eliacik, Umut Cagiral, Evin Iscan, Gunes Ozhan

**Affiliations:** 1 Hamidiye Faculty of Dental Medicine, Department of Pediatric Dentistry, University of Health Sciences, Istanbul, Turkey; 2 Izmir International Biomedicine and Genome Institute (IBG-Izmir), Dokuz Eylul University, Izmir, Türkiye; 3 Izmir Biomedicine and Genome Center (IBG), Dokuz Eylul University Health Campus, Izmir, Turkey; 4 Department of Molecular Biology and Genetics, Izmir Institute of Technology, Izmir, Turkey; University of Puthisastra, CAMBODIA

## Abstract

Testing the biocompatibility of commercially available dental materials is a major challenge in dental material science. In the present study, the biocompatibility of four commercially available dental materials Mineral Trioxide Aggregate, Biodentine, Harvard BioCal-CAP and Oxford ActiveCal PC was investigated. The biocompatibility analysis was performed on zebrafish embryos and larvae using standard toxicity tests such as survivability and hatching rates. Comparative toxicity analysis of toxicity was performed by measuring apoptosis using acridine orange dye and whole mount immunofluorescence methods on zebrafish larvae exposed to the dental materials at different dilutions. Toxicity analysis showed a significant decrease in survival and hatching rates with increasing concentration of exposed materials. The results of the apoptosis assay with acridine orange showed greater biocompatibility of Biodentine, Oxford ActiveCal PC, Harvard BioCal-CAP and Biodentine compared to MTA, which was concentration dependent. Consequently, this study has shown that showed resin-modified calcium silicates are more biocompatible than traditional calcium silicates.

## Introduction

Vital pulp treatments (VPT), such as pulp capping, are therapeutic strategies that preserve the integrity, and pulpal vitality of teeth with deep carious lesions and trauma or mechanical exposure of the pulp [1]. There are many materials used in pulp capping [2]. An ideal pulp capping material should refrain from an inflammatory pulpal response that could lead to necrosis and promote the formation of reparative dentin [3]. The development of mineral trioxide aggregate (MTA) in 1998 [4] and Biodentine in 2010 [5] has popularized the use of calcium silicate-based materials in dental pulp [6]. MTA and Biodentine have shown reliable and long-term results compared to calcium hydroxide due to their biocompatibility, mechanical properties, and promotion of reparative dentin formation [7–9]. To improve the physical-mechanical properties of MTA while maintaining its biological advantages, light-cured resin modified calcium silicate-based materials have been introduced [10,11]. The introduction of new calcium silicate-based materials has facilitated the effective preservation of healthy pulp tissue [12]. Resin-modified calcium silicate-based materials support the hypothesis of the present study, as they have a lower toxicity profile and higher biocompatibility compared to traditional calcium silicate materials.

Harvard BioCal-CAP (Harvard), launched in 2019 by Harvard Dental International GmbH, Germany [13], and the recently introduced resin-modified calcium silicate material Oxford ActiveCal PC (Oxford) by Oxford Scientific, Germany, for which only one study has been published in the literature [14]. Nevertheless, residual monomers produced during the polymerization of dental materials exhibit cytotoxic effects [15]. It is crucial to investigate the cytotoxicity and biocompatibility of calcium silicates and resin-modified calcium silicates used for pulp capping [16].

The zebrafish, in vivo model, exhibits genetic and physiological similarities with humans is preferred in cytotoxicity studies [17,18]. The cellular, structural, and biochemical similarities between humans and zebrafish will enable the rapid prediction of potential effects of chemicals and other substances on human [19]. In addition to genetic and physiological similarities, zebrafish possess several advantages, including transparency, rapid reproduction and development, ease of maintenance, and cost-effectiveness [20]. These advantages may facilitate an increase in zebrafish studies in the future [21].

In the present study, the toxicity and biocompatibility of four different calcium silicate-based pulp capping agents, considered the gold standard and recently introduced, are evaluated. The null hypothesis of the present study is revealed that resin-modified calcium silicate materials have a lower toxicity profile and higher biocompatibility compared to traditional calcium silicate materials.

## Materials and methods

This animal study has been written according to Animal Research: Reporting of In Vivo Experiments (ARRIVE) guidelines [22]. The present study was ethically approved by Animal Experiments Local Ethics Committee of Izmir Biomedicine and Genome Center (IBG-AELEC) on 09/03/2022 under protocol number 2022–010. The present study was carried out in the Izmir Biomedicine and Genome Center Zebrafish facility. Zebrafish larvae (5 days post-fertilisation, dpf) were used to investigate embryonic developmental toxicity. At the end of the 120-hour period, all apoptosis and Whole-Mount Immunostaining procedures were performed under Tricaine methanesulfonate (MS-222) anesthesia, and euthanasia was conducted using hypothermic shock [23].

### Sample size

G*Power 3.1.9 software was used to determine the minimum sample size required for the study, with a medium effect size (f = 0.25), 95% statistical power, and 0.05 error margin. The total sample size for each group was set at 180 embryos, with 30 embryos for each subgroup.

### Zebrafish maintenance

Adult wild-type zebrafish were maintained in the Zebrafish Facility of Izmir Biomedicine and Genome Center with a 14:10 light/dark cycle at a temperature of 28°C. Fish were fed twice a day with a flake food and Artemia salina [24].

### Preparation of test solutions

MTA Angelus (Angelus, Brazil), Biodentine (Septodont, France), Oxford (Oxford Scientific, Elmshorn, Germany), and Harvard (Harvard Dental International GmbH, Germany) were prepared according to the manufacturer’s instructions. The details pertaining to the materials, including their intended purpose, manufacturer, composition, setting type, and setting time, showed in the Table 1.

**Table 1:** The properties of dental materials: Purpose of usage, manufacturer, composition, setting type and setting time.

Material	Purpose of usage	Manufacturer	Composition	Setting type	Setting time
Biodentine	Bioactive Dentin Repair Material	Septodont, France	Powder: Tricalcium silicate powder Liquid: Calcium chloride and excipients as accelerator	self-hardening	12 minutes
MTA Angelus	Endodontic Repair Cement	Angelus Indústrıa De Produtos Odontológıco Ltda., Brazil	Powder: Tricalcium Silicate, Dicalcium Silicate, Tricalcium Aluminate, Tetracalcium Aluminoferrite, Bismuth Oxide, Iron Oxide, Calcium Carbonate, Magnesium Oxide, Calcium Oxide, Crystalline Silica Liquid: Distilled Water	self-hardening	15 minutes
Harvard BioCal-CAP	Bioactive, Light-Curing Resin-Modified MTA cement for Direct and Indirect Pulp Capping	Harvard Dental International GmbH, Germany	Mixture of mineral oxide species and methacrylates	Light cure using a polymerization unit (wavelength range 400–500 nm) with a light intensity of at least 1000mW/cm2.	40 seconds
Oxford ActiveCal PC	Light Cured Resin Reinforced MTA Pulp Capping Material	First Scientific Dental Materials, Germany	No information has been shared by the manufacturer other than the fact that it contains MTA fillers and is reinforced with resin.	Light cure using a polymerization unit (wavelength range 400–500 nm) with a light intensity of at least 1000mW/cm2.	40 seconds

1 g of each material was placed in 50 ml sterile falcon tubes containing E3 control medium (5mM NaCl, 0,17 mM KCl, 0,33 mM CaCl2, 0,33 mM MgSO4, and 0,1% methylene blue) which facilitated the maintenance the viability of in zebrafish embryos and larvae. The tubes were sterilized under UV for 15 minutes and then incubated at 37°C for 24 hours to ensure the stability of the solution. Each tube was centrifuged for 1 minute and filtered using 0.2 μm filters [25]. For each material group, a serial dilution determined as 1:1, 1:2, 1:4, 1:8, 1:16, and 1:32. Test solutions prepared in 2 ml Eppendorf tubes at the specified dilutions in the table were transferred to six-well cell culture plates.

### Determination of embryonic toxicity

#### Survival and hatching rate

Seven mating tanks, each containing a barrier, were prepared and a total of 21 females and 14 males were placed. The barrier was removed the next day. Approximately 2 hours later, the embryos were collected from breeding tanks where adult zebrafish mate. The zebrafish embryos (n = 30) were transferred to six-well plates containing the prepared solutions. Each well contained a specific material dilution (1:1, 1:2, 1:4, 1:8, 1:16 and 1:32) to ensure consistent exposure. The embryos and larvae exposed to test solutions at different dilutions and were examined at 0, 24, 48, 72, 96, and 120 hours using a stereomicroscope (SZX10 Olympus). The numbers of dead embryos and larvae were recorded, and the solutions were replaced with freshly prepared solutions every 24 hours. The effect of all test groups on larval hatching was quantifies in the 48h-120h interval. The embryos and larvae were incubated at 28°C. The experiment was performed in three replicates times.

### Acridine orange assay

To analysis the apoptotic effects of the test solutions at the 120th hour, larvae were treated with acridine orange. Test groups (n = 10), MTA (1:8, 1:16, 1:132), Biodentine (1:32), Harvard (1:2, 1:4, 1:8, 1:16, 1:32), Oxford (1:4, 1:8, 1:16, 1:32) were treated with acridine orange.

The 1 ml acridine orange (C014 10 mg/mL, 1 mL, ABP Biosciences, ABD) stored at -20°C and dissolved in a DMSO to achieve a dilution of 10 mg/mL was prepared. 2 μL of acridine orange was added to all groups and then larvae were incubated in the dark for 60 min. The larvae were washed three times with E3 medium, and then they were examined under a fluorescent microscope (Olympus SZX16). The florescent intensity of tail was analysed using ImageJ for each group and the results were displayed as a graph.

### Whole-mount immunostaining

To investigate the apoptotic effect of MTA 1:8 and Harvard 1:8 caspase-3 was assesed using the whole mount immunofluorescence. The whole mount immunofluorescence staining of zebrafish larvae were performed as described previously [26].

Larvae were fixed in 4% paraformaldehyde (PFA) in 1X PBS overnight at 4°C. The next day larvae treated with 100% ice-cold methanol at -20°C for overnight.

The next day, larvae were rehydrated with phosphate-buffered saline (PBS) and diluted with dilutions of methanol in a solution containing 0.1% Triton-X-100, and then washed with PBS-0.1% Triton-X-100 solution. Larvae were blocked for 2 hour in PBDX GS blocking buffer (10% bovine serum albumin, 1% DMSO, 0.3% Triton-X, 15 μL/1 mL goat serum) and PBDX_GS was removed and 40μL dilution of primary antibody (Cleaved caspase-3, (5A1E) CST, rabbit) was added and larvae were incubated at 4°C overnight. The next day primary antibody was removed and larvae were washed. PBS containing 0.05% Tween-20 (PBS-T). The next steps were performed in the dark. 100 μL of diluted secondary antibody (Fluorescein (FITC) AffiniPure Goat Anti-Rabbit IgG (H+L) at 1:400 dilution in PBDX_GS) was added. Nuclear staining was carried out using 4′,6-diamidino-2-phenylindole (DAPI; 4083S, Cell Signaling Technology, MA, United States).

Then secondary antibody dilution was removed and washed and larvae were embeded into the 80% glycerol and stored at +4°C in the dark. Larvae were imaged at 25× magnification using a laser confocal microscope (ZEISS LSM 880, ZEISS Group, Germany) and the number of Cleaved-Caspase-3 positive cells were analyzed using the ImageJ program. The results were displayed as a graph.

### Statistical analysis

The results of the study were analyzed using the "GraphPad Prism 8.0.2." software (GraphPad Prism, San Diego, CA). Two-way ANOVA was used to assess the significance of the difference between the groups (materials and dilutions). The symbols used in statistical significance show different levels of significance: p > 0.05 (ns) indicates no statistically significant difference, p ≤ 0.05(*) marginally significance, p ≤ 0.01(**) statistical significance, p ≤ 0.001(***) a high level of statistical significance.

## Results

### The effects of the dental materials on the embryos and the survival rate of the larvae varied

To investigate the biocompatibility of the dental materials MTA, Biodentine, Harvard and Oxford, zebrafish embryos were treated with different dilutions (1:1, 1:2, 1:4, 1:8, 1:16 and 1:32) of the tested materials and examined under a stereo microscope for 24, 48, 72, 96 and 120 hours.

**Fig 1:**
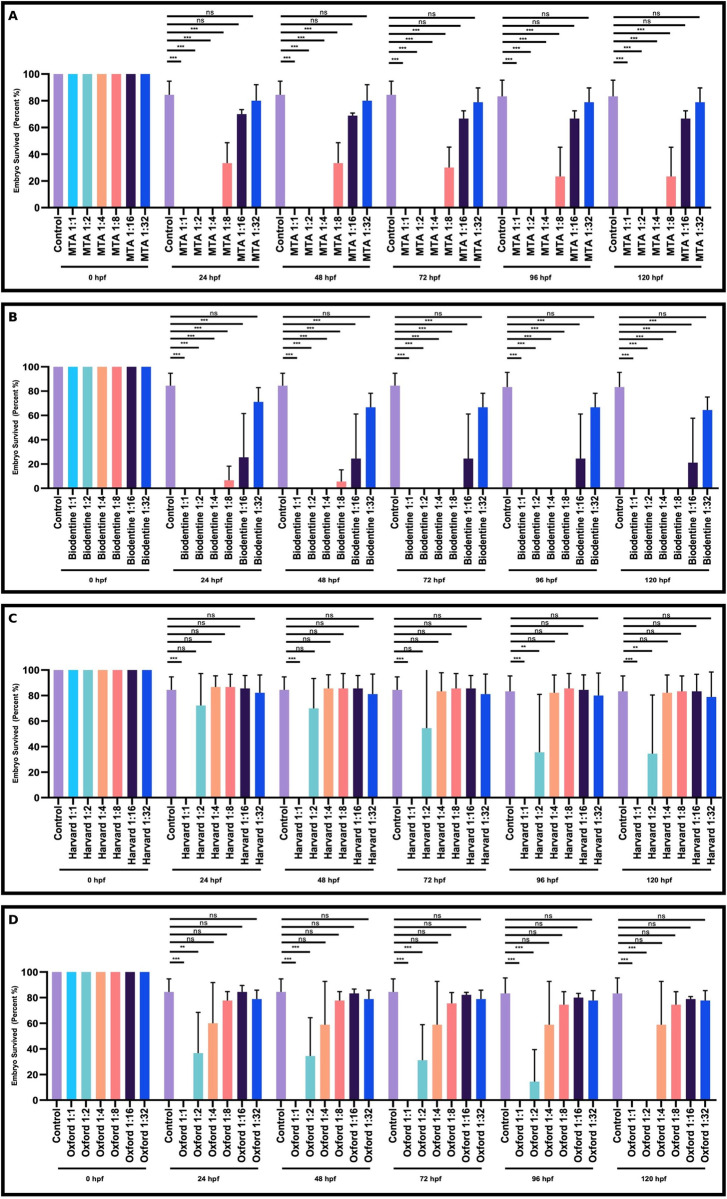
Dose-dependent effect of dental materials on the survival of zebrafish embryos and larvae at the indicated time points. Comparative analysis of the effect of MTA (**A**), Biodentine (**B**), Harvard (**C**) and Oxford (**D**) at 1:1, 1:2, 1:4, 1:8, 1:16, 1:32 on the survival rate of embryos and larvae after 24, 48, 72, 96 and 120 hours of treatment compared to the control group. The columns and error bars show ± SD of triplicate experiments. ns (not significant), ** p < 0.001 and *** p < 0.0001 for significant differences between embryos and larvae treated with the different dilutions of dental materials. (n = 30) for each group).

The survival rate of the embryos was dramatically reduced after 24 hours with a 1:1 dosage of all materials (**Fig 1A–1D**).

MTA significantly reduced the survival rate of the embryos and larvae at the 1:2, 1:4, 1:8 dilutions while the 1:16 and 1:32 dilution had no significant effect on the survival rate (**Fig 1A**).

Biodentine was drastically reduced the survival rate of the embryos and larvae at the 1:2, 1:4, 1:8 and 1:16 dilutions at the indicated time points. The dilution of 1:32 had no significant effect on the survival rate of zebrafish embryos and larvae (**Fig 1B**).

However, Harvard at the following dilutions 1:2, 1:4, 1:8, 1:16 and 1:32 had no significant effect on the survival rate of embryos and larvae at the indicated time points (**Fig 1C**).

In addition, Oxford decreased survival rate of the embryos and larvae 1:2 dilution while the other dilutions did not significantly affect the survival rate of the embryos and larvae at the indicated time points (**Fig 1D**).

These results indicated that the resin-modified calcium silicates materials, Harvard and Oxford materials have a greater effect on the survival rate of zebrafish embryos and larvae than the traditional calcium silicates materials, MTA and Biodentine.

### The tested dental materials reduced the hatching of the larvae in different dilutions and at different time points

**Fig 2:**
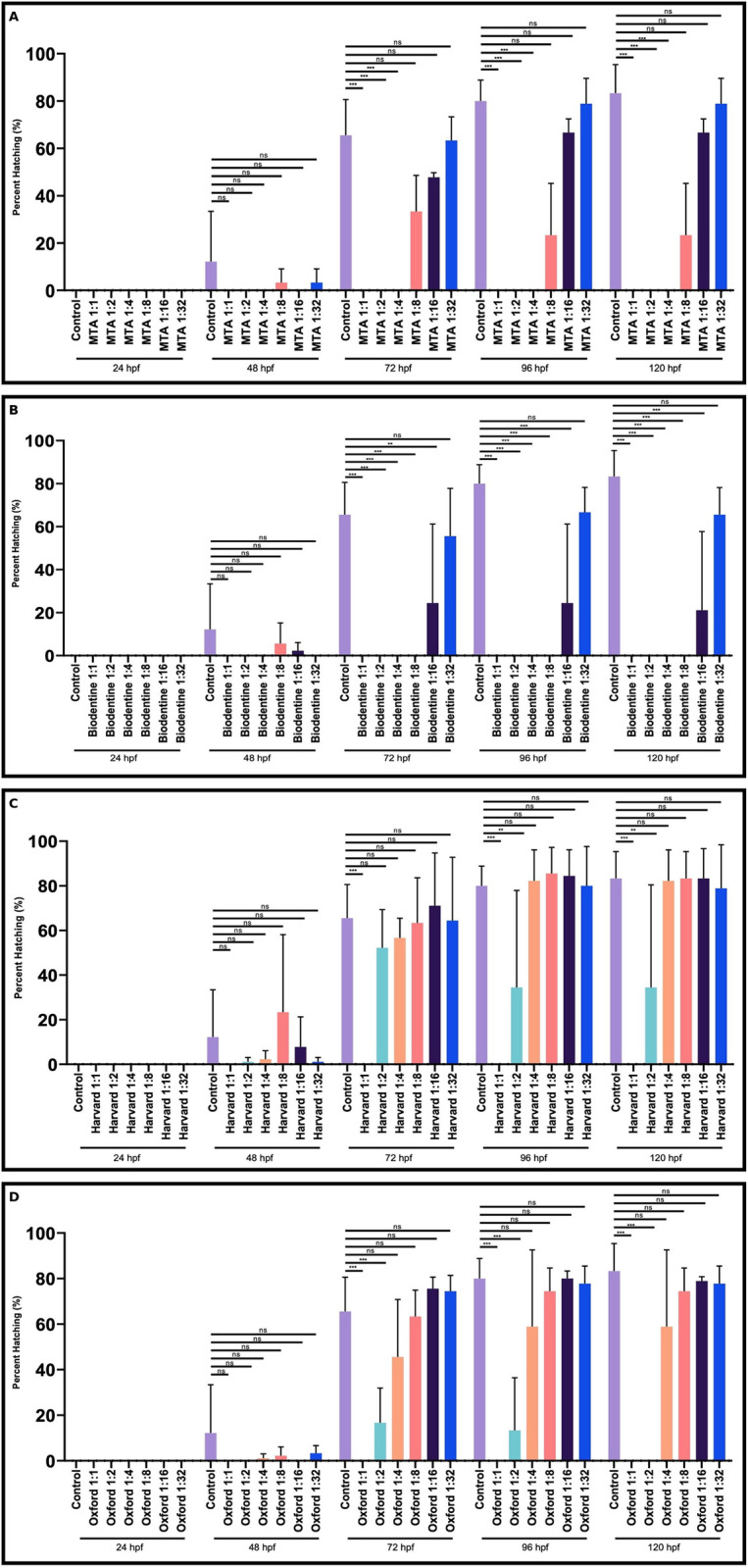
Dose-dependent effect of dental materials on hatching rate of zebrafish embryos and larvae at the indicated time points. Comparative analysis of the dilution of MTA (A) 1:8, 1:16 and 1:32, Biodentine (B) 1:32, Harvard (C) and Oxford (D) 1:2, 1:4, 1:8, 1:16, 1:32 on the hatching rate of larvae after 48, 72, 96 and 120 hours compared to the control group. Columns and error bars indicate ± SD from triplicate experiments. Ns (not significant), ** p < 0.001 and *** p < 0.0001 for significant differences between embryos and larvae treated with the different dilutions of dental materials compared to the untreated control group. ((n = 30) for each group).

To investigate the developmental toxic effects of the dental materials MTA, Biodentine, Harvard, Oxford on the hatching rate of zebrafish larvae after 48, 72, 96 and 120 hours, they were analysed. MTA, Biodentine, Harvard and Oxford had no significant effect on the hatching rate at any dilutions of the materials compared to the control group after 48 hours (**Fig 2A–2D**).

After 72, 96 and 120 hours, the hatching rates were not significantly changed at the MTA dilutions 1:8, 1:16 and 1:32, at which the larvae still survive (**Fig 2A**).

At a dilution of 1:16, Biodentine significantly reduced the hatching rates of the larvae after 72, 96 and 120 hours (**Fig 2B**).

Harvard reduced larval hatching rates at the higher 1:2 dilution after 72, 96 and 120 hours (**Fig 2C**).

Oxford, however, had no effect on larval hatching rates at any of the dilutions at the time points indicated (**Fig 2D**).

These results showed that MTA and Oxford had no effect on hatching rates, while Biodentine at a dilution of 1:16 and Harvard at a dilution of 1:2 reduced larval hatching significantly.

### The tested dental materials induced apoptosis in increasing dilutions

**Fig 3:**
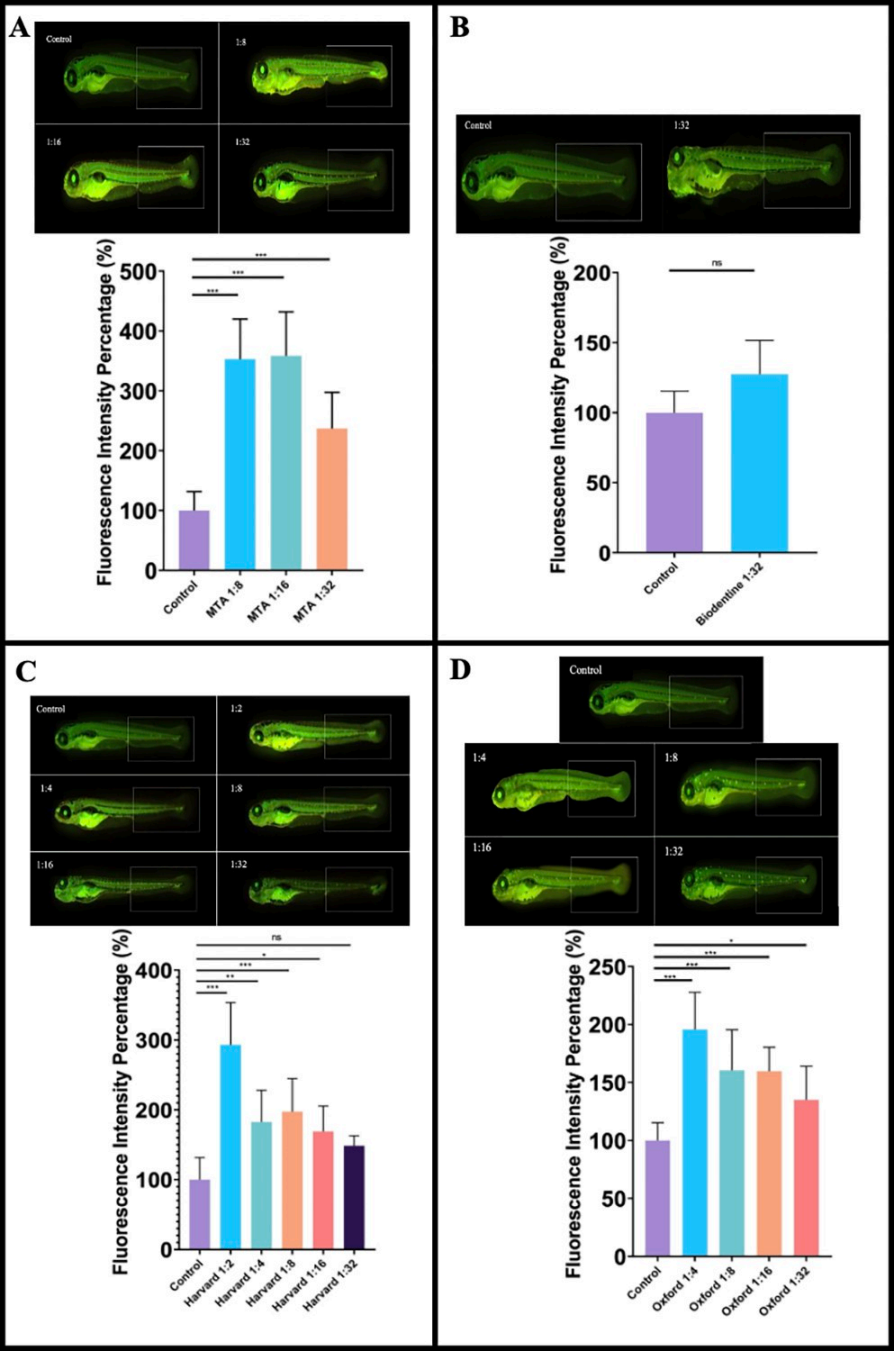
The apoptic effects of dental materials on zebrafish larvae. The fluorescence microscope images of larvae treated with MTA (1:8, 1:16, 1:32) (A), Biodentin (1:32) (B), Harvard (1:2, 1:3, 1:8, 1:16, 1:32) (C) and Oxford (1:4, 1:8, 1:16, 1:32) (D). The fluorescence intensity tails of the larvae were analysed using ImageJ. ((n = 10) for each group). Columns and error bars indicate ± SD from triplicate experiments. Ns (not significant), ** p < 0.001 and *** p < 0.0001 for significant differences between embryos and larvae treated with the different dilutions of dental materials. Scale bars 500μm.

To investigate the apoptotic events induced by the dental materials in the 120th zebrafish larvae, we used an acridine orange, fluorescent dye. The fluorescence intensity was measured in the caudal region in the posterior part of the zebrafish larvae (**Fig 3A–3D**).

MTA at dilutions of 1:8, 1:16 and 1:32 significantly increased apoptosis (**Fig 3A**).

Biodentine at a dilution of 1:32 had no effect on apoptosis (**Fig 3B**).

Harvard enhanced apoptotic responses at the higher dilutions 1:2, 1:4, 1:8 and 1:16, the lower dilution 1:32 had no significant effect on apoptosis (**Fig 3C**).

Oxford also significantly induced apoptosis at the higher dilutions 1:4, 1:8 and 1:16, while the dilution 1:32 induced apoptosis less significantly (p ≤ 0.05) (**Fig 3D**)

**Fig 4:**
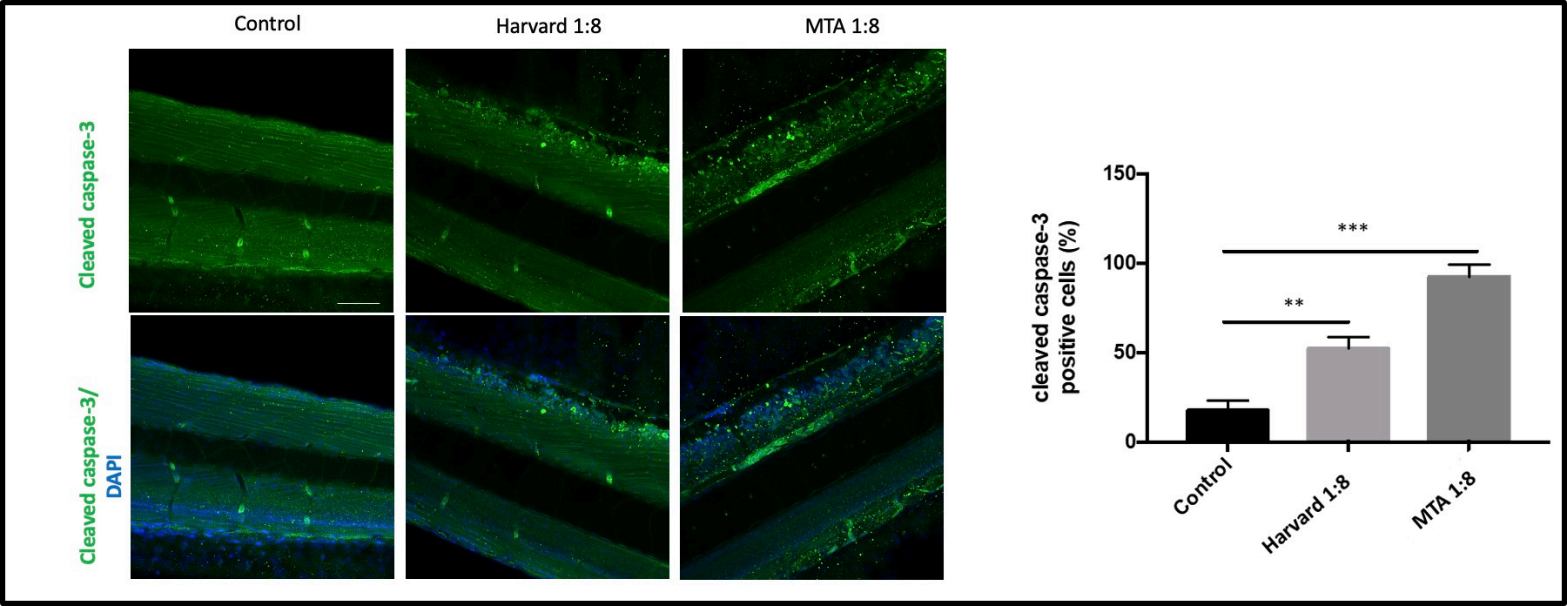
Determination of Cleaved Caspase-3 positive cells in MTA and Harvard treated groups. The florescence images of Cleaved Caspase-3 positive cells in the control group larvae and larvae treated with MTA 1:8 and Harvard 1:8. DAPI (blue) was used for nuclear counterstain. Images were taken at 25× magnification in a confocal microscope. ((n = 10) for each group). Scale bar Columns and error bars indicate ± SD from triplicate experiments. ns (not significant), ** p < 0.001 and *** p < 0.0001 for significant differences between embryos and larvae treated with the different dilutions of dental materials. Scale bars 50 μm.

To confirm the apoptotic responses determined by the acridine orange assay, we performed a whole mount immunofluorescence assay 120 hours after treatment of embryos with Harvard 1:8 (moderately induced apoptosis) and MTA 1:8 (dramatically induced apoptosis). Consistent with the acridine orange assay, whole mount active caspase-3 staining showed that MTA 1:8 treatment dramatically induced apoptosis, while the effect of Harvard 1:8 on cleaved caspase-3 activation was moderate compared to the control group (**Fig 4**).

## Discussion

Resin-modified calcium silicate-based materials demonstrate a lower toxicity profile and higher biocompatibility compared to traditional calcium silicate materials, thus supporting the hypothesis.

Vital Pulp Therapy (VPT) plays a crucial role in the preservation, repair, and extended maintenance of dental pulp tissue within the oral cavity. The use of newly introduced calcium silicate-based materials and advanced treatment strategies has facilitated the efficacious preservation of healthy pulp tissue [12]. These pulp capping materials are utilized to facilitate the maintenance of normal tissue function and vitality, aiming to enable tissue to regain and sustain its normal function. They are expected to enable repair of the damaged tissue efficiently and have low toxicity. As new VPT materials enter the market, studies continue to be conducted to investigate the biocompatibility properties of these materials [27,28]. Recent studies have used zebrafish embryos and larval models to investigate the biocompatibility of dental materials [29]. In the present study, the toxicity and biocompatibility of four different calcium silicate-based pulp capping agents (MTA, Biodentine, Harvard and Oxford), considered the gold standard and recently introduced, are evaluated on zebrafish embryos and larvae. The biocompatibility analysis was performed using standard toxicity tests such as survivability, hatching rate and apoptosis.

Survival rates of zebrafish embryos and larvae varied when exposed to MTA, Biodentine, Oxford, and Harvard materials at different dilutions (1:1, 1:2, 1:4, 1:8, 1:16, and 1:32). MTA Angelus and Biodentine materials exhibit significantly low survival rates at higher doses (1:1, 1:2, and 1:4). Even at the lower doses of MTA 1:8, Biodentine 1:8, 1:16, the survival rate of embryos and larvae was significantly reduced at the indicated time points (Fig 1A and 1B). In contrast, even at high dilutions, Oxford and Harvard materials demonstrate higher survival rates compared to other groups. However, a previous study conducted in cell culture has shown that Biodentine did not negatively affect cell viability and proliferation, whereas higher dilutions exhibited cytotoxic effects [30].

Resin modified calcium silicates materials, Harvard and Oxford reduced the survival rate of embryos and larvae survival rates at the higher 1:1 and 1:2 concentrations, but at the lower 1:8, 1:16 and 1:32 doses, Harvard and Oxford had no effect on embryos and larvae survival at the different time points (Fig 1C and 1D). These findings are consistent with previous research; for instance, a study conducted on rats reported that resin-modified calcium silicates show less biocompatibility and have a reduced bioinductive effect compared to traditional calcium silicates [31]. However, in a study examining the regeneration and biocompatibility of the dentin-pulp complex in a mouse model, both resin-modified and traditional calcium silicates were found to be biocompatible [32]. Another in vitro study examining TheraCal LC and traditional calcium silicates on stem cells’ viability, proliferation, and differentiation found that all materials are biologically compatible and support cell proliferation while maintaining viability [33]. These results highlight the complexity of material interactions and the need for further research to understand the varying responses in different experimental models.

Another important indicator for the assessment of toxicity is the hatching rate. Zebrafish embryos hatch 48 hours after fertilization. In the present study, the hatching rates of zebrafish embryos were examined after treated with different concentrations of MTA, Biodentine, Harvard and Oxford. Our results showed that even at the lower doses MTA 1:8 and Biodentine 1:16 decreased hatching times, while Harvard and Oxford only hatching rate at the higher dose 1:2 (Fig 2A–2D). Previous research has demonstrated that different doses of perfluorooctanesulfonate cause a decrease in embryo hatching rates [34], and zinc oxide nanoparticles synthesized using papaya extract have been reported to delay hatching [35]. These results highlighted the variability in biocompatibility between different pulp capping materials and different concentration. The present study revealed that increasing concentrations of traditional calcium silicates, Biodentine and MTA, significantly reduced survival and hatching rate of embryos and larvae and they were less biocompatible than resin-based calcium silicates, Harvard and Oxford.

In the present study, the effect of four dental materials on the apoptotic responses of zebrafish embryos and larvae were analyzed by acridine orange, fluorescent dye. High concentrations of the materials increased the apoptotic response, but at low concentrations, there was no statistically significant difference compared to the control group. Resin-modified calcium silicates showed better results than traditional calcium silicates, demonstrating lower apoptotic responses. Our results showed that the resin- modified calcium silicates are more biocompatible than traditional calcium silicates. These findings align with previous studies, which have shown that traditional and resin-based calcium silicate materials do not induce apoptosis in in vitro cell culture [36,37]. However, a study conducted in 2016 demonstrated that resin-modified calcium silicates induce a higher apoptotic response [38]. Additionally, studies on zebrafish embryos examining developmental toxicity with acridine orange and Cleaved Caspase-3 have also reported an increase in apoptosis [39,40]. These results highlight the variability in apoptotic responses based on material type and concentration, underscoring the need for further research to fully understand these effects.

Consequently, this in vivo study demonstrated the superior biocompatibility of resin-modified calcium silicates compared to traditional calcium silicates, holding significant clinical relevance. These findings suggest a potential shift in the selection of materials used in vital pulp treatment procedures, potentially improving treatment outcomes. However, the present study has limitations, including the need for a larger sample size to enhance statistical power. Additionally, clinical studies are recommended to further validate the cytotoxicity and biocompatibility of these pulp capping materials. Although the study addresses developmental toxicity, further research is required to evaluate toxicity and biocompatibility in adult zebrafish.

## Conclusion

In conclusion, resin-modified calcium silicate materials (Oxford and Harvard) are found to be less toxic and more biocompatible even at higher concentrations compared to traditional calcium silicate materials (MTA Angelus and Biodentine). Further in vitro and in vivo studies are needed on resin-modified calcium silicate materials.
